# Extended Low Temperature and Cryostorage Longevity of *Salix* Seeds with Desiccation Control

**DOI:** 10.1515/biol-2019-0001

**Published:** 2019-03-20

**Authors:** Ja Jung Ku, Sim Hee Han, Du Hyun Kim

**Affiliations:** 1 Department of Life Resources Industry, Dong-A University, Busan 49315, Republic of Korea; 2 Department of Forest Genetic Resources, National Institute of Forest Science, Suwon 16631, Republic of Korea

**Keywords:** Seed germination, Salicaceae, *Salix xerophila*, *Salix maximowiczii*, *Salix koreensis*

## Abstract

*Salix xerophila*, *S. maximowiczii*, and *S. koreensis* are species of willow native to Korea that are important for bioenergy production. However, the native range of these species has narrowed in recent years due to the impact of climate change. Seeds of these *Salix* species lose viability within 4 weeks at ambient temperature, and within 4 months at -4°C. Preservation techniques are urgently needed to protect these valuable resources. The effects of seed water content (SWC; 3%, 6%, 9%, 12%, 18%, and 24%) and temperature (ambient, 4°C, -18°C, -80°C, and -196°C) on storage stability were investigated for up to 48, 52, or 60 months, depending on species. Optimal storage temperature and SWC varied between species. *S. xerophila* seed could be stored without deterioration for 60 months with 9% SWC at -80°C, but rapidly lost viability when stored at -18°C. In *S. maximowiczii* and *S. koreensis*, 100% and 90% of normal germination, respectively, was maintained with 18% SWC at -18°C or -80°C. Thus, for some *Salix* species, storage at -18 and -80°C may provide an economical alternative to cryopreservation or medium-term storage for the maintenance of seedbanks or breeding stocks.

## 1 Introduction

Many *Salix (willow)* species are characterized by their rapid growth and high biomass as well as their ability to tolerate adverse conditions such as flooding and their capacity to accumulate heavy metals, organic pollutants, and pollutants caused by eutrophication [1, 2]. The increasing severity of global crises caused by severe environmental pollution and a reduction in the availability of energy resources has increased the economic importance of *Salix* species, given their utility for a wide array of practical applications such as phytoremediation [1] and bioenergy production [2, 3].

Revegetation of *Salix* plantations is usually achieved by planting cuttings. However, seed propagation is used to maintain genetic and sexual diversity for conservation as well as propagation purposes. The most challenging aspects of *Salix* seedling propagation are the collection of seeds in the wild, and seed storage [3].

The shelf life of willow seeds varies greatly depending on species. Salicaceae species generally lose seed viability over the medium term when stored at 4°C. Seeds from these species were found to be desiccation intolerant, recalcitrant, or of intermediate classification with respect to seed storage, since dried seeds lost viability within months of storage at -20°C [3, 4, 5, 6, 7, 8]. According to Ayi *et al*. [9], production of *S. variegate*, an important species for riparian protection in reservoir regions, was limited by the rapid loss of seed viability that constrained seedling production. Bonner [10] described seeds from *Salix* species as suborthodox, because seeds could be stored in orthodox conditions only for short time periods. Seeds of a fall-dispersing Alaskan willow species, *S. glauca*, retained higher viability than seeds from summer-dispersing willow species after 36 months of hermetic storage at -10°C [11], and seeds of 24 common *Salix* species from the Alaskan boreal forest and tundra retained viability for several years [12]. Seeds of *S. rehderiana ×* (*S. × capreola*), *S. × sericans × S. viminalis*, *S. alba*, and *S. matsudana* were found to be desiccation tolerant and could be dried to approximately 5–10% moisture content (fresh weight) [13-15]. Germination viability in seeds of summer-dispersing willows (*S. alaxensis*, *S. bebbiana*, and *S. novae-agliae*) decreased by 20–40% after 36 months of storage [11]. Storage parameters such as seed water content (SWC) and temperature influence seed longevity. Maroder *et al*. [14] showed that seed viability of *S. alba* decreased as storage temperature increased (-20°C, 2°C, and 16°C) when seeds were stored for 150 days. Similarly, viability of seeds from two *Salix* hybrids, desiccated to 5% and 10% SWC and stored for 68 days, improved as storage temperature decreased (-196°C, -20°C, 2°C, and 16°C) [15].

Cryopreservation can be used to extend storability of seed from several *Salix* species as liquid nitrogen (LN) storage is thought to slow aging and preserve viability indefinitely. Cryostorage was therefore presumed to be the only wholly effective option for long-term conservation of *Salix* seeds, and optimal SWC for LN storage was investigated in several previous studies [14, 15, 16]. Dry seeds of *S. alba* [14], *S. rehderiana ×* (*S. × capreola*) [15], *S. caprea* [17], *S. gracilistyla* [16] , and *S. bebbiana*, *S. exigua*, *S. myricoides*, and *S. petiolaris* [18] were successfully cryopreserved with SWC of 4.3–22%. However, Ballesteros and Pence [18] reported that aging was not completely stopped during cryostorage and, for some *Salix* species, seed longevity was shorter than predicted.

Thirty *Salix* species are native to the Korean Peninsula, including *S. xerophila*, *S. maximowiczii*, and *S. koreensis*. *S. xerophila*, a deciduous broadleaved tree species, is distributed in the North province of South Korea, Mongolia, Russia, and Europe. *S. xerophila* grows in rocky areas near the top of mountains and disperses its seeds in the summer. *S. maximowiczii*, a deciduous broadleaved tree species, is distributed in the North province of South Korea, Russia, China, the Far East, and East Asia. *S. maximowiczii* grows in valleys of elevated regions, grows to a maximum height of 15 meters, and disperses its seeds in June–July. *S. maximowiczii* can be used for silvicultural applications such as forest erosion control, timber production, and development of green river bank areas. *S. koreensis* grows to a maximum height of 20 meters and is native to Korea, Japan, and China. These three species are of interest in Korea for the production of bioenergy, and studies are underway to improve their breeding. However, the natural ranges of these species are shrinking as a result of climate change, and there is an urgent need to develop effective preservation technologies to protect these natural resources. However, limited information is available regarding storage protocols for *Salix* species, and mechanical freezing has only been examined for short storage periods or for a limited range of *Salix* species. Storage conditions for *S. xerophila*, *S. maximowiczii*, and *S. koreensis* have not been determined previously.

The objective of this study was to examine differences in seed viability associated with storage conditions for *S. xerophila*, *S. maximowiczii*, and *S. koreensis* and specifically to investigate (1) whether *Salix* seed viability can be retained over storage periods of 48–60 months in a mechanical freezer, (2) how storage temperature and SWC affect seed storability, and (3) whether SWC ranges can be identified for LN storage.

The results presented in this study are important for developing protocols for medium- and long-term storage of seeds from *Salix* species using mechanical freezing and cryopreservation methods.

## 2 Materials and Methods

### 2.1 Plant materials, catkin collection, and seed cleaning

Catkins were collected when cotton emerged from partially opened capsules. Catkins of *S. xerophila* Flod. were collected from natural forests in Mt. Odae (37°48´N 128°33´E, 1257 m elevation, Gangwon Province, Republic of Korea) on June 14, 2012. Catkins of *S. maximowiczii* Kom. were collected from Mt. Hamback (37°12´N 128°54´E, 1235 m elevation, Gangwon Province, Republic of Korea) on June 27, 2013, and *S. koreensis* Andgersson catkins were collected from Mt. Duckyoo (35°55´N 127°42´E, 374 m elevation, Jeonam Province, Republic of Korea) on May 8, 2013. Catkins were collected from several trees growing within a 50 meter area with no other willow species located nearby, to limit the potential for cross-pollination. Samples were transported to the laboratory and processed immediately. To open capsules fully, catkins were arranged in a single layer and dried for 2 days at ambient temperature or in an incubator for 2 days. Seeds were cleaned from the cotton using air and soil screens as described by Dreesen [4].

### 2.2 Desiccation of seed and storage conditions

After catkin collection, but before processing, fresh seeds had SWC of 32.9% and 42.5% in *S. xerophila* and *S. koreensis*, respectively. SWC of *S. maximowiczii* was not measured, since catkins were not sufficiently open to allow separation of seed upon collection.

Catkins were placed in an open dish to dry in an incubator at 20°C under light or were stored at 4°C in a plastic bag (without sealing) for 2 days to maintain SWC. After catkins opened at 20°C, seeds were cleaned from cotton and 400 mg seed samples placed over 100 g fresh silica gel or 90 ml distilled water in sealed plastic containers (110 × 110 × 35 mm) for desiccation and rehydration, respectively, and kept at 20°C. Seed water content was monitored during desiccation and hydration treatments and determined gravimetrically [19] for samples weighting 20 mg each (~300 seeds) after drying at 130 °C for 1 h. Seeds dried with silica gel to 3%, 6%, 9%, 12%, 18%, and 24% SWC were then sealed in double aluminum bags within a polythene bag and stored at several temperatures: RT (~25°C), 4°C, -18°C, and -80°C.

*S. maximowiczii* and *S. koreensis* seeds were used to evaluate seed longevity at RT (~25°C). Seeds were stored at RT for 4 and 5 weeks for *S. maximowiczii* and *S. koreensis*, respectively.

The effects of low temperature mechanical cooling (4°C, -18°C, and -80°C) were investigated using seeds from all three *Salix* species. Germination was evaluated after storage periods of 1, 4, 12, 24, 36, 48, and 60 months for *S. xerophila*; 5, 12, 24, 36, and 52 months for *S. maximowiczii*; and 5, 12, 24, 36, and 48 months for *S. koreensis*. Seeds stored in freezers at -18 and -80°C were warmed on a closed Petri dish at room temperature for at least 20 minutes prior to germination testing.

For cryopreservation, seeds were placed in 2 ml plastic cryovials (Nalgene, USA) which were sealed and plunged directly in LN. After 1 week of storage at -196°C, ampoules were rewarmed in a water bath at 37°C for 90 s. Optimal storage conditions were defined as a range of which ensured > 80% maximum survival (normal germination) before storage in consideration of SWC and temperature.

### 2.3 Seed germination test

Seeds were placed on top of two layers of filter paper moistened with distilled water in 90 mm Petri dishes at 20 ± 1°C under 24 h constant light. Germination was checked daily for 7 days, and assessed when normal and abnormal seedlings could be easily distinguished. Seedlings with cotyledons, hypocotyl, and roots were considered to be normal. For each combination of seed moisture content, temperature, and storage duration, germination was assessed in four replicates containing 25 seeds.

### 2.4 Statistical analysis

Statistical analyses were conducted using SAS software (SAS Institute, USA). ANOVA was used to assess the influence of storage temperature, SWC, and storage period on normal germination following an arcsine transformation. Means were compared using the Duncan multiple range test (DMRT) at the 5% level. Significant differences between non-treated (-LN) and cryopreserved (+LN) seeds were compared using Student’s t-test.

**Ethical approval**: The conducted research is not related to either human or animals use.

## 3 Results

### 3.1 Germination of *Salix* spp. seeds during storage at room temperature

**Figure 1:**
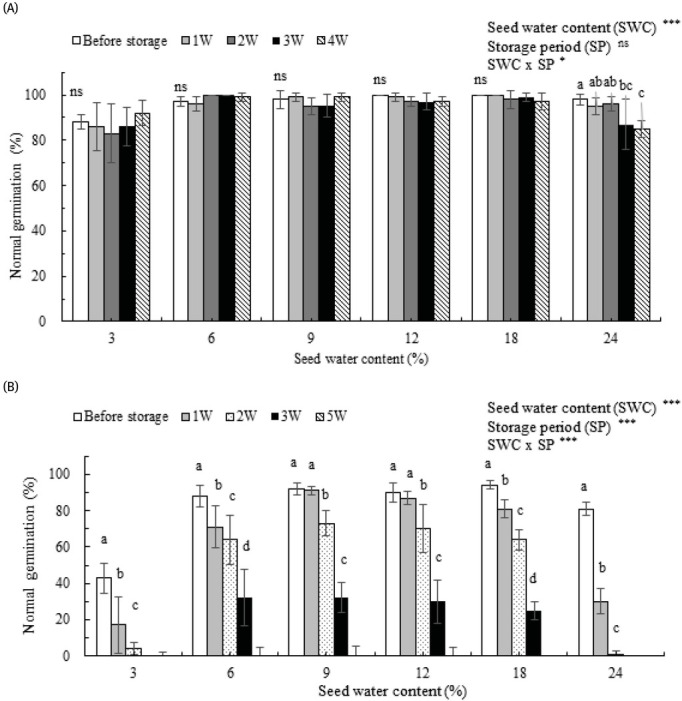
Normal germination of seeds before storage (0 W) and after 1–5 weeks (1 W–5 W) of storage at room temperature in *Salix maximowiczii* Kom (A) and *S. koreensis* Andersson (B). Seed water contents were 3%, 6%, 9%, 12%, 18%, and 24% (fresh weight basis). Different letters indicate statistical differences among five storage durations within each seed water content at p < 0.05, according to Duncan’s multiple range test. Vertical bars represent SD of the mean (n=4). *** p < 0.001, * p < 0.05, and ns: non-significance.

After seed cleaning by air separation, 87%, 100%, and 97% of seeds germinated from *S. xerophila*, *S. maximowiczii*, and *S. koreensis*, respectively. Desiccation and moisture influenced seed viability in the three *Salix* species. Reducing SWC to 3% significantly decreased normal germination in all three species (Figure 1 and Table 1).

**Table 1:** Effects of seed water content (SWC) and cryopreservation (LN) on normal germination of *S. xerophila*, *S. maximowiczii*, and *S. koreensis* seed.

Seed water content Normal germination (%) (SWC, %)						

	*S. xerophila*		*S. maximowiczii*		*S. koreensis*	

	-LN	+LN	-LN	+LN	-LN	+LN
After seed cleaning			100		97	
87						
3	68 ± 3.3^abz^	61 ± 9.8^b^	88 ± 3.3^b^	91 ± 6.8^ns^	43 ± 8.2^c^	47 ± 6.8^c^
6	69 ± 3.8^a^	64 ± 11.0^ab^	97 ± 2.0^a^	100 ± 0.0	88 ± 5.7^ab^	91 ± 8.9^ab^
9	72 ± 7.3^a^	77 ± 10.0^a^	98 ± 4.0^a^	100 ± 0.0	92 ± 3.3^ab^	93 ± 3.8^a^
12	61 ± 5.0^b^	54 ± 13.7^b^	100 ± 0.0^a^	100 ± 0.0	90 ± 5.2^a^	89 ± 8.2^ab^
18	53 ± 5.0^c^	39 ± 5.0^c*^	100 ± 0.0^a^	100 ± 0.0	94 ± 2.3^a^	95 ± 5.0^a^
24	51 ± 5.0^c^	31 ± 8.2 ^c*^	98 ± 2.3^a^	99 ± 2.0	81 ± 3.8^b^	83 ± 9.5^b^
Significance						
SWC	p > 0.001		p > 0.001		p > 0.001	
LN	p > 0.001		ns		p > 0.001	
SWC × LN	p > 0.001		ns		p > 0.001	

Values are means of four replicates ± SD (n=4). Within columns, values with the same letter are not significantly different at p=0.05 with Duncan’s multiple range test. * indicates significant difference between non-treated (-LN) and cryopreserved (+LN) seeds. ns: non-significance.

Seed storability at room temperature (RT) was examined for *S. maximowiczii* and *S. koreensis*, and seeds from the two species exhibited different storage stabilities at RT with respect to SWC. *S. maximowiczii* seeds exhibited no significant differences in normal germination with SWC of 3%, 6%, 9%, 12%, and 18% after 4 weeks of storage at RT. However, seeds with 24% WC exhibited significantly reduced viability (13% germination) after 4 weeks of storage compared with germination before storage (p > 0.05) (Figure 1A). Normal germination in *S. koreensis* decreased significantly with increased storage periods at RT for all SWCs (p > 0.05). Seeds with 9% SWC exhibited the slowest deterioration over time: germination decreased by 1% after storage for 1 week, 20.6% after 2 weeks of storage, 65.2% after 3 weeks of storage, and 100% after 5 weeks of storage (Figure 1B). These data suggest that *S. maximowiczii* seeds survive better than those of *S. koreensis* when stored at RT, and are also tolerant to a wider range of SWC (Figure 1).

### 3.2 Effects of water content and storage temperatures on seed viability in three *Salix* spp

**Figure 2:**
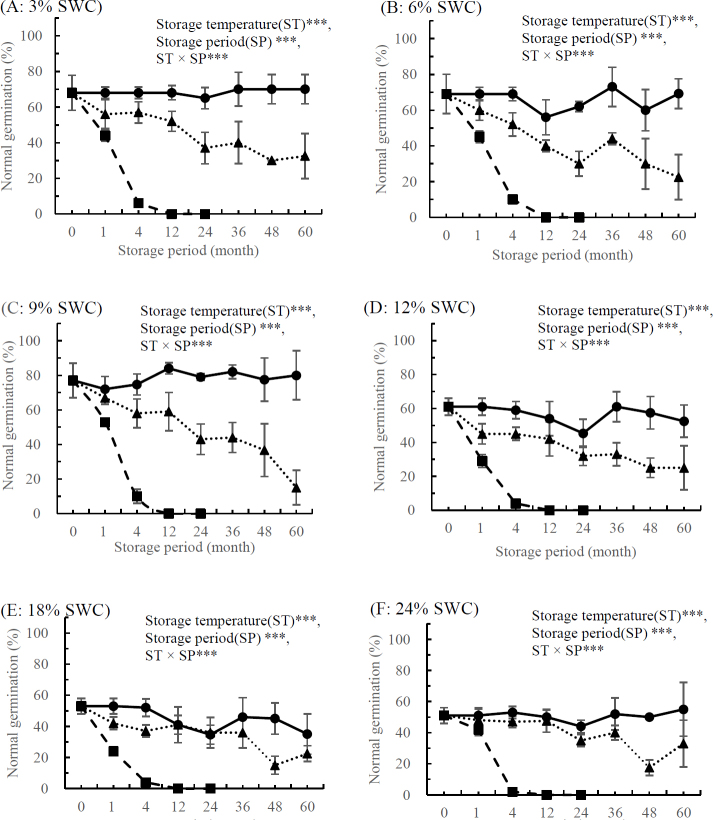
Effects of storage temperature at 4°C (■), -18°C (▴), and -80°C (●) on normal germination in S. xerophila Flod. Seeds were stored for up 60 months with water contents controlled to 3% (A), 6% (B), 9% (C), 12% (D), 18% (E), or 24% (F) (fresh weight basis). Storage temperature (ST)***, seed water content (SWC)***, storage period (SP)***, ST × SWC***, ST × SP***, SWC × SPns, ST × SWC × SP**. All values are means of four replicates ± SD (n=4). ** and *** indicate significant difference at p < 0.01 and p < 0.001, respectively, and ns indicates non-significance.

**Figure 3:**
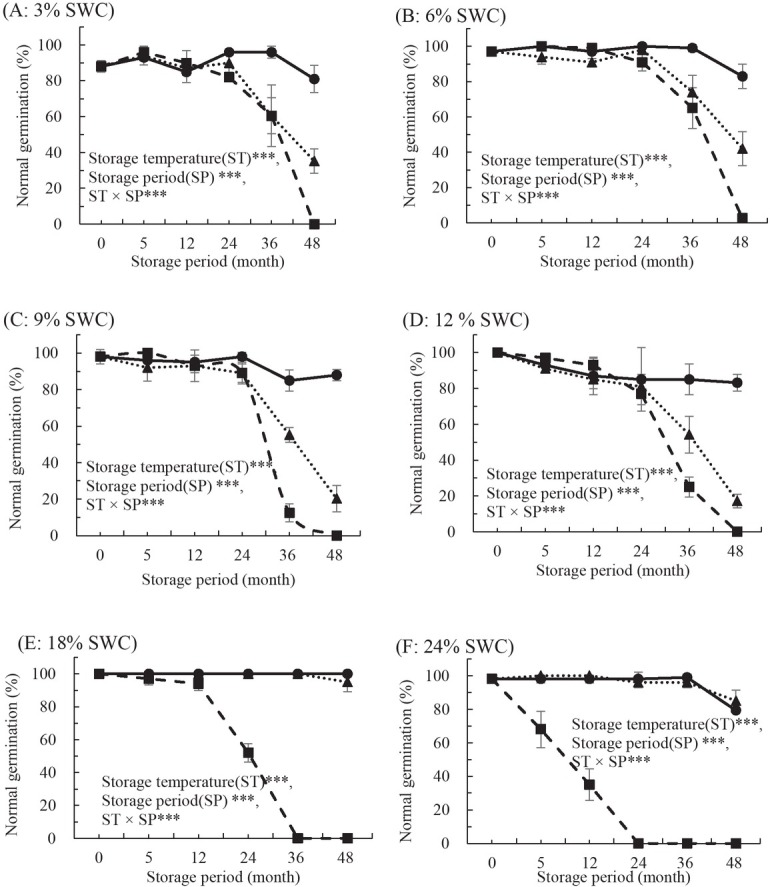
Effects of storage temperature at 4°C (■), -18°C (▴), and -80°C (●) on normal germination in *S. maximowiczii* Kom. Seeds were stored for up 48 months with water contents controlled to 3% (A), 6% (B), 9% (C), 12% (D), 18% (E), or 24% (F) (fresh weight basis). Storage temperature (ST)***, seed water content (SWC)***, storage period (SP)***, ST × SWC***, ST × SP***, SWC × SP***, ST × SWC × SP***. All values are means of four replicates ± SD (n=4). *** indicates significant difference at p < 0.001.

Multivariate analysis of variance (ANOVA) revealed significant effects of storage temperature, SWC, storage period, and their interactions on normal germination (p > 0.001). Significant effects were seen for all interactions across the three species, except between SWC and storage period in *S. xerophila* (Figures 2–3). The effects of SWC on seed longevity were investigated after storage at 4°C, -18°C, and -80°C for *S. xerophila* (Figure 2). For 4°C and -18°C, seed longevity declined after 1 month of storage for all SWCs. Normal germination declined dramatically after 4 months of storage at 4°C (average 91% loss for six different SWCs) and -18°C (average 26% loss for six different SWCs). After 60 months of storage at -18°C, normal germination decreased by 35–81% (average: 59% loss), depending on the SWC content, compared with germination before storage. By contrast, seeds stored at -80°C exhibited no decline in normal germination after 60 months of storage compared with germination before storage (Figure 2).

Experimental series were conducted to compare the effects of SWC and storage temperature on seed longevity in *S. maximowiczii* (Figure 3). Five of six SWCs showed >85% germination after 12 months of storage at 4°C, -18°C and -80°C. The only exception was SWC of 24%, which exhibited 35% normal germination after 12 months at 4°C. Longevity varied among seeds with different SWC at 4°C storage. Germination rates for seeds with 3% and 6% SWC decreased to 61% and 65% after 36 months of storage at 4°C, compared with 87% and 97% germination prior to storage, respectively. By contrast, normal germination in seeds with 9%, 12%, 18%, and 24% SWC decreased to 0–25% after 36 months of storage. Normal germination in seeds with all SWCs dropped to zero after 48 months of storage at 4°C. After 48 months of storage at -18°C, viability of seeds with 3%, 6%, 9%, and 12% SWC decreased dramatically by 69%, 57%, 79%, and 83% compared with germination prior to storage. Seeds with 18% and 24% SWC exhibited germination declines of only 5% and 13%, respectively. No loss in viability was observed in seeds stored at -80°C until after 36 months of storage, at which point germination decreased by 0–19% for all SWCs when germination rates before storage were compared with those after 48 months of storage.

**Figure 4:**
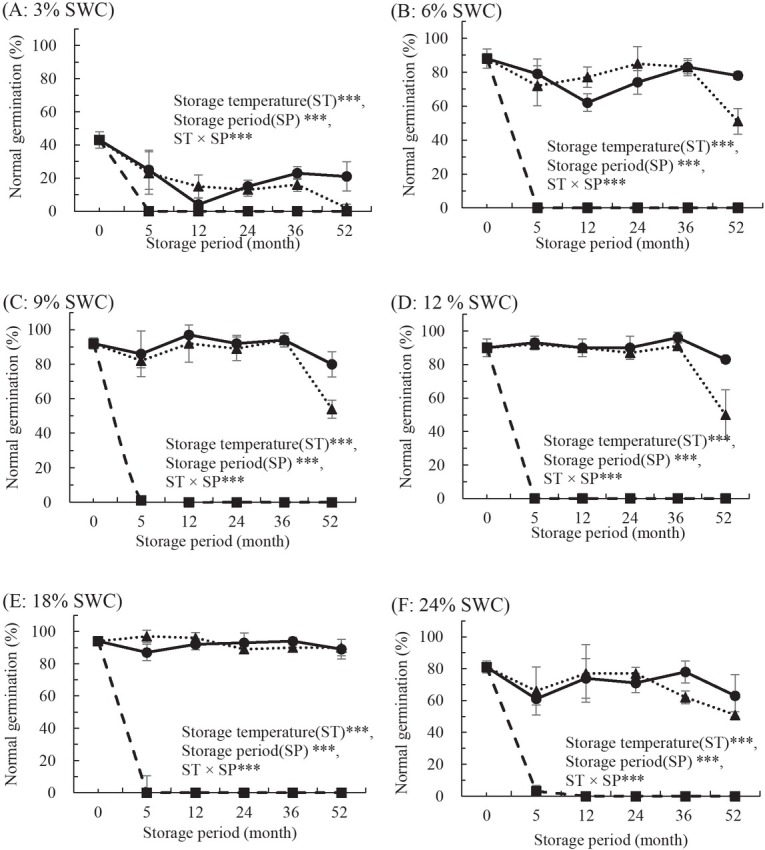
Effects of storage temperature at 4°C (■), -18°C (▴), and -80°C (●) on normal germination in *S. koreensis* Andersson. Seeds were stored for up 52 months with water contents controlled to 3% (A), 6% (B), 9% (C), 12% (D), 18% (E), or 24% (F) (fresh weight basis). Storage temperature (ST)***, seed water content (SWC)***, storage period (SP)***, ST × SWC***, ST × SP***, SWC × SP***, ST × SWC × SP***. All values are means of four replicates ± SD (n=4). *** indicates significant difference at p < 0.001.

*S. koreensis* seed germination was also significantly affected by storage temperature, SWC, storage period, and the interactions between these factors (p > 0.001). In *S. koreensis*, viability of seeds stored at 4°C dropped to zero after 5 months of storage, whereas seeds stored at -18°C and -80°C exhibited no decline until after 36 months of storage, with the exception of seeds with 3% and 24% SWC (Figure 4). After 52 months of storage, viability decreased to 4–51% compared with pre-storage in seeds with 3%, 6%, 9%, 12%, 18%, and 24% SWC stored at -18°C. However, seeds with 6%, 9%, 12%, or 18% SWC showed minimal viability losses (5–13%) when stored at -80°C, whereas viability losses were more pronounced in seeds with 3% and 24% SWC (73% and 22%, respectively) (Figure 4).

### 3.3 Effects of seed water content and cryopreservation on *Salix* spp. seed viability

Freshly collected seeds of three *Salix* species, S. *xerophila*, *S. maximowiczii*, and *S. koreensis*, were tested for their response to desiccation and subsequent cryopreservation. Storing *Salix* seeds cryogenically in LN was found to be effective and feasible. For all three species, normal germination was unaffected when seeds were cryopreserved within their safe SWC range, as determined by comparison of germination rates in non-LN treated (-LN) vs. cryopreserved seeds (p > 0.05). Based on 95% binomial confidence intervals, survival of seeds at 18% and 24% SWC decreased after LN_2_ treatment compared with non-LN treated seed (-LN) in *S. xerophila*, whereas seeds with SWC 3-12% exhibited no adverse effects (Table 1). Table 2 summarizes the storable period and germination rate according to storage temperature and SWC to clarify the optimal storage conditions that ensure> 80% normal germination rate before storage. LN_2_ treatment had no effect on normal germination in *S. maximowiczii* and *S. koreensis* in seeds with 3–24% SWC, however, defining normal germination conditions as a range that ensures > 80% of normal germination before

**Table 2:** Summary of optimal seed storage conditions for each species of *P. davidiana* and *P. koreana*.

Storage			Species			
temperature (°C)	*S. xerophila*		*S. maximowiczii*		*S. koreensis*	

	Optimal SWC range (%)	Storage period (months); NGP(%)	Optimal SWC range (%)	Storage period (months); NGP(%)	Optimal SWC range (%)	Storage period (months); NGP(%)
After seed cleaning	87%		100%		97%	
RT	NA	NA	3-24	1; 85-99%	9-18	0.25; 81-91%
4	NA	NA	3-6	24; 82-91%	NA	NA
-18	NA	NA	18-24	48; 85-95%	6-18	36; 83-94%
-80	3-9	60; 70-80%	3-24	48; 80-100%	6-18	52; 78-89%
-196	9	NA; 77%	3-24	NA; 91-100%	6-24	NA; 83-95%

Normal germination percentage (NGP); Room Temperature (RT); Seed water content (SWC); Not available (NA)

storage, 9%, 3-24% and 6-24% of SWC is recommended for LN storage in *S. xerophila*, *S. maximowiczii* and *S. koreensis* (Table 2). These results emphasize the importance of further characterizing the effect of SWC on seed viability and elucidating safe SWC ranges for preservation for each *Salix* species.

## 4 Discussion

Under natural moisture and temperature conditions, seeds from *Salix* species are short-lived in storage [12, 14-17, 20]. In this study, seeds from *S. koreensis* completely lost viability within 5 weeks at RT (Figure 1B). Similar results were obtained for *S. koreensis* by Brinkman [21], who concluded that seeds of North American *Salix* species had to be sown within 4 to 6 weeks after collection because seeds could not be stored. In accordance with the reported short life of *S. alba* seeds, viability was lost in 2 weeks at 25°C [14]. The viability of *S. variegata* seeds decreased very quickly as seeds were viable for only 9 days and mostly germinated within 3 days if dispersed to a suitable environment [22]. Regardless of the timing of seed production (October, November, or December) or temperature applied to stored seeds (5–30°C), seed viability did not exceed 16 days, and seeds produced in October retained viability for only 8 days [9, 21].

Seed storability at RT can vary depending on *Salix* species. In our results, no significant reductions in normal germination rates (>97% germination) were observed for *S. maximowiczii* after 4 weeks of ambient storage at 6-18% SWC range (Figure 1A and Table 2). Seeds of both *S. koreensis* and *S. maximowiczii*, which possessed high SWC, showed further decreases in viability when stored above 0°C (RT and 4°C). Consistent with this, Popova *et al*. [16] reported that *S. gracilistyla* seeds with lower SWC were more viable after 40 days of storage than were seeds without desiccation. In previous studies, the maximum storage period of viable seeds from *Salix* species varied from a few days to several months at ambient temperature [12, 14, 15, 23, 24].

This study confirmed that seeds from *S*. *xerophila* and *S. koreensis* were in the short-lived category of seeds from *Salix* species, as dried seeds from these species lost viability almost completely within 4–5 months of storage at 4°C. Based on previous results with seeds of hybrid and autumn willows [14, 15], and on our preliminary studies with *S. caprea*, *S. gracilistyla*, and *S. hallaisanensis* [16, 17], it was expected that the *Salix* seeds in this study would lose viability most rapidly after approximately 8–10 weeks of storage time at 4°C. Storage at 5°C for approximately 10 weeks significantly reduced the viability of seed from a wild species and from three *S. caprea* clones, while viability of seeds from four additional clones remained unaffected [17]. It was therefore expected that the three *Salix* species considered in this study would exhibit different viability responses. The seed viability of *S. xerophila* and *S. koreensis* decreased significantly within 16 and 20 weeks of storage at 4°C, respectively, while the viability of *S. maximowiczii* remained unchanged (Figures 2, 3, and 4). These results were consistent with those of previous studies that noted the impacts of storage environment (temperature and moisture) and species on seed longevity [25-27] and later seedling growth from stored seeds [28]. Similar results were observed for *S. maximowiczii* seeds, where freshly harvested seed initially showed no signs of aging until a threshold time was reached, after which viability was rapidly lost. Longevity can be influenced by moisture and storage temperature as well as by seed traits influenced by growth environment and genetics [29]. The seed storability of *P*. *davidiana and P*. *koreana* species differed based on species, and their optimum storage conditions were different. The optimum storage conditions of the two species showed significant differences according to SWC and storage temperature [30]. Therefore, as well as species, seed SWC strongly affected storage longevity of seeds from *Salix* species when stored above 0°C. Seeds from all three species survived up to 48–60 months of storage at -80°C, and a wider range of SWC was tolerated at this temperature than at 4°C or -18°C. Our analysis of long-term stored seed viability confirmed that storage temperature and SWC significantly affected normal seed germination capacity in three *Salix* species. This was consistent with previous reports showing that storage at sub-zero temperatures increased seed longevity to 6 or even 12 years but was damaging to their viability [26, 31, 32]. Germination of seeds from four *Salix* species stored for 36 months at -10°C declined from 96% to 75% [11]. Optimal SWC for *S. hallaisanensis* and *S. gracilistyla* seed storage was reported to be 8–10% [16], and Simpson and Daigle [33] observed that seed moisture contents between 5% and 10% did not have an adverse impact on storability in *S. bebbiana*, *S. discolor*, and *S. eriocephala* in sub-zero temperature storage.

Our results showed that the rate of decreasing viability was greatest for seeds stored at RT, followed by those stored at 4°C, -18°C, and -80°C. Relatively long-term storage of *Salix* seeds from different species at sub-zero temperatures was reported previously [11, 14, 33, 34, 35]. For example, seed of *S. matsudana* at -70 °C maintained initial germination levels for up to 30 months, and viability of *S. alba* seeds was extended for several weeks at 5°C and for several months at -20°C [14]. In contrast, European beech (*Fagus sylvatica* L.) seed with 8-9 % SWC can be stored for 10 years without loss of germination at -5 to -10 °C. In this case although seeds of European beech belong to the sub-orthodox, there were no significant difference occurring in germination between beechnuts stored at –7°C or –22°C for 3–4 years [36]. In our results, the relatively short seed longevity at -18°C compared with -80°C could be due to differences between species as noted in previous studies, which precludes strict comparisons of results between studies [14, 16].

In the present study, no adverse effects on normal germination were observed after LN immersion (cryostorage), with the exception of *S. xerophila* seeds with SWC of 18% and 24%. Although seed viability varied with SWC in each *Salix* spp., SWC of ~9% was optimal in all three species. This was consistent with results from Maroder *et al*. [14], which showed that dry seeds (4.3–12% SWC) of *S. alba* and *S. matsudana* survived immersion in LN without loss of viability, and that seed from two *S. caprea* clones at 11% SWC also survived cryopreservation with high viability [17]. By contrast, survival of seeds with 10% SWC decreased with increasing storage time at all temperatures, whereas seeds with 5% SWC lost no viability when stored for 2 months in LN_2_ [15]. In *S. caprea* seeds [17], the range of safe SWC varied significantly depending on the clone and vigor of seed, and was wider for seeds with the highest viability (5–22% SWC) compared with the lowest vigor seeds (17–27% SWC). In the present study, considering the overall germination rate change with SWC before cryostorage, the safe SWC range was narrowed in *S. xerophila*. and *S. koreensis* seeds compared to the other species. As a result, species and seed vigor may be factors that may affect the safe SWC range for cryopreservation of Salix species, as has also been reported in *Populus* species [30, 37]. LN storage extended the viability of seeds with short lifespans in comparison with the standard temperatures of *Salix* seed banks. However, deterioration occurred during cryogenic storage faster than expected, particularly for seeds of initial low quality [18]. It was reported that, within a short time frame (two decades), aging reactions progressed during LN storage and, depending on the accession, significant decreases in normal germination and seed survival, or even complete loss of survival, were seen [18, 24].

Low temperatures and SWC are important factors in the storage of *S. xerophila*, *S. maximowiczii*, and *S. koreensis* seed. Storage of seeds at 4°C was not effective for maintaining normal seed germination in the three *Salix* species. Storage at -18°C was not universally effective, but cryopreservation or storage at *-*80°C gave acceptable longer-term storage results for all three species in this study. Therefore, determining the optimal SWC range for each species is critical for successful long-term storage. Although storage at -18°C was not suitable for storage of *S. xerophila*, 18% SWC seeds of *S. maximowiczii* and *S. koreensis* could be stored for 48 months and 36 months without deterioration, provided the seeds were within their safe SWC storage range. When seeds were stored at -80°C, seed from all three *Salix* species retained viability for up to 48–60 months with a wider range of SWC. Thus, seed desiccation to 9% SWC prior to cryopreservation can be recommended for these three species. In addition, the range of suitable SWCs for cryogenic storage for these three species is broader than -80 °C storage (Table 2).

In conclusion, mechanical freezer storage at -18°C and -80°C presents a viable alternative to cryopreservation for longer term storage of *Salix* seeds. Freezer storage can be an easier procedure to maintain a seedbank of these species, but this work demonstrated that storage in LN is safer for long-term conservation according to all parameters evaluated (see Table 1). Thus, for some *Salix* species, storage at -18 and -80°C may provide an economical alternative to cryopreservation or medium-term storage of maintenance of seedbanks or breeding stocks.
